# Tumor spheroids and organoids as preclinical model systems

**DOI:** 10.1515/medgen-2021-2093

**Published:** 2021-12-03

**Authors:** Aria Baniahmad

**Affiliations:** Institut für Humangenetik, Universitätsklinikum Jena, Am Klinikum 1, 07740 Jena, Germany

**Keywords:** tumor model systems, three-dimensional cancer model, Cancer organoids, patient-dervied xenograft

## Abstract

The generation of three-dimensional (3D) cancer models is a novel and fascinating development in the study of personalized medicine and tumor-specific drug delivery. In addition to the classical two-dimensional (2D) adherent cell culture models, 3D spheroid and organoid cancer models that mimic the microenvironment of cancer tissue are emerging as an important preclinical model system. 3D cancer models form, similar to cancer, multiple cell–cell and cell–extracellular matrix interactions and activate different cellular cascades/pathways, like proliferation, quiescence, senescence, and necrotic or apoptotic cell death. Further, it is possible to analyze genetic variations and mutations, the microenvironment of cell–cell interactions, and the uptake of therapeutics and nanoparticles in nanomedicine. Important is also the analysis of cancer stem cells (CSCs), which could play key roles in resistance to therapy and cancer recurrence. Tumor spheroids can be generated from one tumor-derived cell line or from co-culture of two or more cell lines. Tumor organoids can be derived from tumors or may be generated from CSCs that differentiate into multiple facets of cancerous tissue. Similarly, tumorspheres can be generated from a single CSC. By transplanting spheroids and organoids into immune-deficient mice, patient-derived xenografts can serve as a preclinical model to test therapeutics *in vivo*.

Although the handling and analysis of 3D tumor spheroids and organoids is more complex, it will provide insights into various cancer processes that cannot be provided by 2D culture. Here a short overview of 3D tumor systems as preclinical models is provided.

## Tumor development

During tumor evolution, preferentially those cellular pathways and cascades that inhibit cell proliferation are progressively downregulated or inactivated. This includes pathways of cell elimination, such as apoptosis, or cell cycle arrest by quiescence or cellular senescence. These three cellular pathways are part of the natural embryogenesis of mammalian development [1], [2]. It was shown that during tumorigenesis, premalignant tumors have higher levels of apoptosis or cellular senescence, which are progressively reduced in malignant tumors [3]. However, the pathways of apoptosis and cellular senescence might be enforced and reactivated in a tumor by drug treatment, thus being a target for cancer therapy. Also, a higher DNA mutation rate and the occurrence of aneuploidy are associated with tumor evolution leading to a more malignant cancer. An increase in mutation load can be an indicator for treatment with PARP inhibitors. In general, tumor evolution is associated with inactivation of tumor suppressors, activation of proto-oncogenes, and stabilization of telomere length by reactivation of telomerase activity or the ALT pathway. Altogether, alterations of these factors and pathways are essential in tumorigenesis and tumor evolution.

Cancer metastasis is a major challenge and a life-threatening event. The dissociation of tumor cells from the primary tumor and the migration, invasion, and transport through the lymphatic system and blood vessels, with the subsequent exit from vessels and generation of distal tumors, are essential steps in metastasis. Metastases also require neo-angiogenesis to grow. Neo-angiogenesis is induced by secretion of pro-angiogenic factors, which stimulate neighboring endothelial cells and induce vessel sprouting. Newly formed tumor blood vessels facilitate the growth of micrometastases by oxygen and nutrient supply. Subsequently, these grow towards the tumor to produce vessels, supporting the new micrometastasis with nutrients and oxygen, allowing the tumor to grow further.

## Cancer stem cells

A major drawback in cancer therapy is the occurrence of therapy resistance, which might evolve during therapy or years after successful treatment. Presumably cancer stem cells (CSCs) play a key role in late cancer recurrence since many drugs target fast growing cancer cells, while CSCs can remain quiescent for a long period of time. In this view, chemotherapy preferentially eliminates the rapidly dividing cancer cells and not or only insufficiently CSCs. These non-affected CSCs may start with cell divisions at a much later time point. The CSC theory is based in part on the observation that only few self-renewal cells are sufficient to generate different tumor cell lineages [4]. CSCs can be quiescent or actively undergo cell divisions. Thereby, the CSCs divide asymmetrically into a CSC and a “normal” cancer cell, which can then undergo apoptosis. The longevity of CSCs may explain why tumors are detected years after apparently successful therapy.

## Structure of cancer

A solid malignant tumor consists not only of cancer cells and CSCs but is a complex structure of cancer and non-cancer cells. The latter includes stroma cells and cancer-associated fibroblasts, immune cells, and lymphatic and vascular endothelial cells, as well as adipocytes [5]. Although these cells may not be neoplastically transformed, they influence the tumor microenvironment, tumor activity, such as proliferation, invasion, and metastasis, and the success of cancer treatment.

Many important insights into tumorigenesis and cancer signaling have been obtained in two-dimensional (2D) cell culture of cancer-derived cell lines. Adherent cell culture is mostly achieved using cells derived from solid tumors. However, the 2D cell culture system does not include important factors of the extracellular scaffold such as matrix proteins, glycoproteins, glycans, and secretion of growth factors, cytokines, and exosomes into the microenvironment. Under most 2D cell culture conditions, the oxygen and glucose concentrations are much higher than inside a solid cancer. An oxygen gradient is better recapitulated by a three-dimensional (3D) tumor cell culture model. All in all, 3D tumor cell models reflect the actual tumor microenvironment better than 2D models.

In general, 3D tumor cell models are analyzed as tumor spheroids, tumorspheres, and tumor organoids for different approaches, including analysis of intratumoral interactions, treatment efficacy, drug delivery, nanomedicine, and individualized medicine.

Especially the analysis of therapeutics in a 3D cell model is a very important field, since cells within the 3D tumor may react differently to the treatment compared to 2D cultured cells due to the interaction with neighboring cells in the 3D microenvironment. Some therapeutics induce apoptosis or cellular senescence in tumors. Senescent tumor cells are irreversibly arrested in the cell cycle, which is beneficial for cancer therapy. However, these senescent cells have a high metabolic activity and secrete chemokines and cytokines, known as the senescent-associated secretory phenotype. On the one hand, these secreted factors may lead to immune cell attraction and immune killing of senescent cancer cells. On the other hand, the senescent-associated secretory phenotype influences neighboring non-senescent cells and induces proliferation within the tumor microenvironment. Therefore, it would be useful to identify compounds that induce apoptosis in senescent tumor cells. This novel research field focuses on identifying senolytic compounds that preferentially target senescent cells for their elimination. Similarly, the elimination of senescent-like CSCs that are potentially immortal is an important approach in cancer therapy.

3D tumor types can be generated and analyzed not only *in vitro*, but also *in vivo* using xenografts in immune-deficient mice to analyze the cancer-specific microenvironment, cancer-specific interactions with neighboring tissues, cancer-related pathways, or therapeutic interventions [6].

## Spheroid tumor model systems

**Figure 1:**
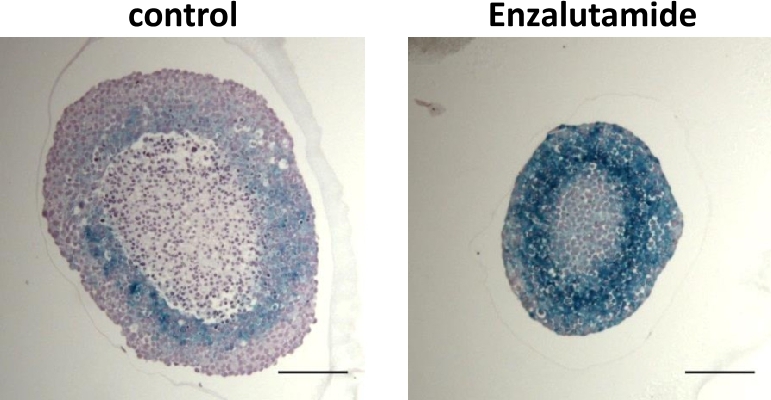
Prostate cancer spheroid treated with the anti-tumor therapeutic androgen receptor antagonist Enzalutamide, which reduces growth. Spheroids from the castration-resistant human prostate cancer cell line C4-2 were generated. After 3 days, spheroids were further cultured for 14 days treated with the second-generation androgen receptor antagonist Enzalutamide or solvent control. Spheroid volume was reduced by antagonist treatment. Spheroid slices were generated and stained for the senescence marker senescence-associated beta-galactosidase and with hematoxylin for nuclear staining. Scale bar: 200 µm.

Spheroids are 3D cell aggregates. Mostly, these are generated by immortalized or transformed tumor-derived cell lines using conventional cell culture media. One can classify spheroids based on the type of used cell line or the use of co-cultures as well as based on the protocol for their generation and cultivation. After generation and growth of spheroids, these cell aggregates differentiate into several layers. The outer layer of spheroids consists of rapidly growing and Ki-67 positive cells. Towards the center of spheroids, the tumor cells exhibit a quiescent, senescent, and necrotic/apoptotic cell phenotype. As an example, treatment with a second-generation androgen receptor antagonist induces the formation of a layer of senescent cells in prostate cancer spheroids (Fig. 1). Also, markers of adhesion can be detected in spheroids. While most tumor spheroids are generated from one cancerous cell line, co-cultures with two or more cell lines can be used to better resemble tumor tissues. Dependent on the scientific hypothesis it is also possible to use co-cultures of a cancerous cell line together with an immortalized cell line or even in combination with primary, non-immortalized cells such as fibroblasts, cancer-associated fibroblasts, or endothelial cells. Co-cultures may change drug penetration or the response to drugs and may exhibit a more resistant phenotype against treatment.

Most screens, especially high-throughput screens for anti-cancer agents, are performed in 2D cell culture models, which is much less time consuming and far cheaper. 3D model systems are much more elaborate and complicated, not only to generate but also to analyze, leading to much higher costs. Thus, for a high-throughput screening, 2D culture remains beneficial. However, 3D cultures could be used subsequently after having narrowed down the number of suitable drug candidates, since 3D tumor model systems are useful to analyze the chemical and physical barriers and the efficacy of compounds in 3D spheroids and organoids prior to the use of animal models [7]. Spheroids have been generated from many cancer types, including breast, ovarian, endometrial, prostate, pancreas, and colon cancers [5], [8], [9], [10]. For prostate cancer androgen-dependent and castration-resistant spheroids were generated. Treatment with anti-cancer compounds reduces growth and may induce cellular senescence as the underlying pathway (Fig. 1).

Several factors play a role that should be considered in order to analyze the efficacy of compounds. The efficacy varies depending on the ability of the compound to bind and act at the outer layer of the spheroids, to penetrate into the inner layer, and to enter tumor cells. For drug penetration into the inner layers of a 3D cell model it is required to consider the spheroid morphology, the inner milieu such as the pH, and the barriers of cell density and interconnectivity of cells through direct cell–cell contact, as well as the extracellular matrix and the ratio of cell types within a co-culture spheroid [11]. Nevertheless, a comparison between 2D and 3D cell culture models has not yet paved the way towards the establishment of a clear picture of which system is more sensitive to analyze chemotherapeutics.

## Tumor spheroid models in nanomedicine

Synthetic nanoparticles represent a promising vehicle tool to deliver drugs. Controlled transport and docking of nanoparticles at the tumor cell are key events for precision medicine and drug delivery [12], [13]. In contrast to 2D cultures, the heterogeneity of tumor tissues may hinder a target-oriented treatment. In addition, metabolism, pH, and oxygen levels are different in tumor tissues compared to normal tissues and 2D cultures. Therefore, it seems more suitable to analyze nanoparticle delivery by taking advantage of similarities of a tumor tissue with 3D tumor spheroid or organoid models. Similar to tumor tissues, the penetration of nanoparticles is hindered by different barriers in 3D spheroids. Direct cell–cell contacts, adhesion molecules, the extracellular matrix, interstitial fluid, and small particles secreted by tumor cells into the spheroid represent barriers for proper nanoparticle-mediated drug delivery. Thus, after an efficacy examination in 2D model systems it might be helpful to analyze the effects of nanoparticles in a 3D model [13], [14].

## Tumorspheres

A special 3D spheroid model system is the tumorsphere. The generation of tumorspheres is mediated by single cancer cells that proliferate in a microenvironment that consists of extracellular matrix, collagen, or methylcellulose cultured with a special stem cell medium. Some cell populations contain CSCs in low abundance. These CSCs can generate cell colonies being embedded in such a matrix from a single cell. Based on their size, the cell colonies are named holo- or meroclones, which represent 3D tumorspheres. These tumorspheres are enriched in CSCs. Increased expression of stemness markers such as CD133 and CD44, as well as key stemness regulators such as Nanog, can be detected. Also, ALDH, which is another important stemness factor, exhibits increased expression. ALDH plays presumably an important role in detoxification and therefore might have an impact on the inactivation of anti-cancer drugs, especially chemotherapeutics [6]. Also, an increase in DNA repair activity is observed in tumorspheres.

The tumorigenicity and malignancy of tumorspheres can be measured using xenografts in immunosuppressed mouse model systems. Since it is suggested that tumorspheres are enriched in cells with stemness characteristics, these tools allow the analysis of CSC activity. However, the enrichment of CSCs in tumorspheres may be a reason why tumorspheres do not share certain histological similarities with tumor tissues [6]. Nevertheless, tumorsphere models can be used to analyze cancer stemness, tumor evolution, and drug resistance.

## Tumor organoids

Patient-derived tumor samples are a promising tool for many areas of tumor biology [15], [16]. The advantage of organoids is that they recapitulate histological and genetic features of the original tumors. Organoids allow the analysis of tumor composition, cell–cell interactions, changes of non-cancer cells by the cancer cell, tumor evolution, the effects of drugs, and drug delivery [17]. Tumor organoids are composed of cancer cells, including CSCs and senescent and apoptotic cancer cells, as well as non-cancer cells such as endothelial cells, cancer-associated fibroblasts, immune cells, and adipocytes. CSCs allow self-renewal and self-organization, which promote malignancy of the tumor. Therefore, tumor organoids allow the analysis within the natural tumor context and microenvironment. This includes the direct cell–cell contact of cancer cells with neighboring cells, the secretome of cancer cells, and their influence on non-cancer cells within the tumor.

The use of tumor organoids in cell culture systems, as well as large-scale biobanking of tumor organoids from genetically predisposed individuals, also permits the testing of therapeutics. For example, organoids from donors with hereditary adenomatous polyposis syndrome caused by pathogenic variants in the adenomatous polyposis coli tumor suppressor gene were studied for the response to all-trans retinoic acid [9].

Further, very good progress was achieved in the generation and analysis of ovarian-derived organoids, especially with high-grade ovarian cancer, which is the most lethal type of ovarian cancer. Ovarian tumor organoids grow quickly in culture and therefore provide a good model system for rapid and personalized testing of drugs as well as combinations of drugs, allowing a better prediction of the response for individual patients [18]. Established organoid lines showed patient tumor-dependent morphology and disease characteristics and recapitulated the parent tumor’s marker expression and mutational landscape [19]. Moreover, organoids displayed tumor-specific sensitivity to clinical high grade serous ovarian cancer chemotherapeutic drugs [20].

PARP inhibitors kill tumor cells with mutations in genes encoding proteins that are involved in double-stranded break repair. High-grade ovarian cancers with pathogenic variants of breast cancer gene 1 (*BRCA1*) and *BRCA2* often respond to PARP inhibitors. Novel classes of therapeutics, such as ATR and CHK1 inhibitors, may be effective in non-responders to PARP inhibitors. However, it would be helpful to predict which patients will have a positive response. Genomic testing for the general mutation load in tumor cells is currently used in order to predict sensitivity to DNA damage repair drugs. However, these may not accurately predict the DNA repair capacity of high-grade ovarian cancers. Therefore, patient-derived organoids as a rapid test platform might be helpful as *ex vivo* pretesting systems in order to predict which patient might benefit from a specific treatment [21], [22].

Recent studies suggest that patient-derived ovarian cancer organoids capture the mutational landscape and histological cancer subtypes of primary tumors, being applicable for drug sensitivity and resistance testing [23], [24]. Those ovarian-derived organoids that harbor a pathogenic *BRCA1* variant exhibit a higher sensitivity to the PARP inhibitor olaparib and to platinum drugs compared to other organoids. The data suggest that patient-derived organoids are suitable physiological *ex vivo* cancer models that can be used to screen effective personalized ovarian cancer drugs effectively.

Recently, organoid biobanks were established to store tissues from donors with *BRCA* germ cell mutations [23] and from individuals affected with pancreatic cancer or neuroendocrine neoplasms [25], [26]. After stratification, these organoid biobanks may allow further analysis such as identification of biomarkers or therapy resistance prediction analysis [27]. In general, the patient-derived organoid research requires larger numbers of samples to validate and standardize the reliability of tumor organoids and the response to treatment.

## Patient-derived xenografts

The use of immune-suppressed mouse strains allows the transplantation of human tumor-derived spheroids and organoids. These 3D cell aggregates can be transplanted as xenografts, e. g., subcutaneously or into the paralogous tissue of origin. Patient-derived xenografts (PDXs) are organoid tumor samples isolated from patients and xenotransplanted into immune-suppressed mice. PDXs can be used preclinically, such as for anti-tumor drug testing, or for analysis in an *in vivo* environment [28]. The advantage of using patient-derived organoids in mice is the possibility to analyze the unchanged tumor itself as well as to test drugs and drug resistance *in vivo*. Although this technique is elaborate, it offers an approach to personalized medicine [29].

## Remarks

**Figure 2:**
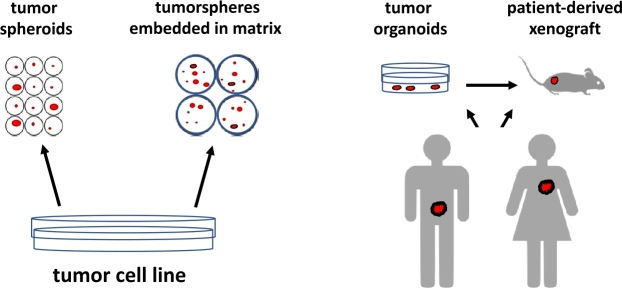
Schematic view of 3D cancer models. Tumor spheroids and tumorspheres can be generated from cancer cell lines. Tumorspheres are embedded in a matrix resembling extracellular matrix which in combination with specific culture media will enrich for stemness in tumorspheres. Tumor organoids preserve patient-specific phenotypic and genetic characteristics and fill the experimental gap between cancer cell lines and animal models in an *ex vivo* system. Preclinical patient-derived xenograft models are preferably used for anti-cancer treatment *in vivo*.

3D tumor spheroids, tumorspheres, and organoids (Fig. 2) are and will be very useful tools to analyze the complexity of cancer cells within a tumor, the intratumoral interaction of cells, the *in vivo* tumorigenicity, and the response to anti-tumor compounds if patient-derived tumor organoids are transplanted into immune-suppressed mice. Also, mechanisms and optimization of drug delivery in complex cancerous tissues will be an important future perspective.
